# A wolf in sheep's clothing—aortic stenosis and cardiac amyloidosis: “RAISE”ing awareness in clinical practice

**DOI:** 10.3389/fcvm.2024.1323023

**Published:** 2024-02-23

**Authors:** H. Sabbour, K. Al-Humood, Z. Al Taha, I. Romany, H. Haddadin, D. Mohty

**Affiliations:** ^1^ Heart and Vascular Institute, Cleveland Clinic, Abu Dhabi, United Arab Emirates; ^2^ Warren Alpert School of Medicine, Brown University, Providence, RI, United States; ^3^ Advanced Heart Failure and Transplantation Unit, Chest Disease Hospital, Kuwait City, Kuwait; ^4^ Sheikh Shakhbout Medical City, Abu Dhabi, United Arab Emirates; ^5^ Pfizer Gulf FZ LLC, Dubai, United Arab Emirates; ^6^ Heart Center, King Faisal Specialist Hospital, Riyadh, Saudi Arabia

**Keywords:** aortic stenosis, diagnosis, cardiac amyloidosis, low-flow/low-gradient, RAISE score, transthyretin, heart failure

## Abstract

Aesop's fable of the wolf in sheep's clothing encourages us to look beneath the exterior appearance of a situation and evaluate the truth that lies beneath. This concept should be applied when managing older patients with severe aortic stenosis. This population of patients is increasingly being identified as having concomitant cardiac amyloidosis, which is an underrecognized cause of common cardiac conditions. The presence of cardiac amyloidosis negatively affects the outcome of patients with aortic stenosis, these patients undergo transcatheter aortic valve replacement (TAVR) with increasing frequency and have a significantly higher overall mortality rate than patients with aortic stenosis alone. Although left ventricular wall hypertrophy is expected in patients with aortic stenosis, it should not be assumed that this is caused only by aortic stenosis. A suspicion of cardiac amyloidosis should be raised in patients in whom the degree of hypertrophy is disproportionate to the degree of aortic stenosis severity. The remodeling, age, injury, systemic, and electrical (RAISE) score was developed to predict the presence of cardiac amyloidosis in patients with severe aortic stenosis. This article highlights the value of increased clinical suspicion, demonstrates the use of the multiparameter RAISE score in daily clinical practice, and illustrates the scoring system with case studies. In elderly patients being considered for TAVR, systematic testing for cardiac amyloidosis should be considered as part of the preoperative workup.

## 1 Introduction

### 1.1 Background to aortic stenosis and cardiac amyloidosis

Aortic stenosis (AS) is the most common valvular heart disease in Western developed countries and a frequent cause of performing a valve procedure (1, 2). The prevalence of AS increases with age, and it is a serious disease in older individuals. AS has been found to be present in >3% of those aged ≥75 and >4% of those aged ≥80 years (1, 3). The pressure overload associated with AS leads to the onset of left ventricular (LV) concentric hypertrophy, impairment of LV diastolic and systolic function, and eventually to heart failure (HF) and death if the aortic valve (AV) is not replaced (1).

Several AS patterns have been described on the basis of the AS valve area, flow, gradient, and left ventricular ejection fraction (LVEF) (2, 4). Severe AS is defined as an AV area of <1.0 cm^2^, generally with a mean transvalvular pressure gradient of ≥40 mmHg. However, a substantial proportion (up to 50%) of patients with AS have low-gradient AS, i.e., a small AV area (<1.0 cm^2^) consistent with severe AS but a low transvalvular pressure gradient (<40 mmHg) consistent with non-severe AS (4).

Low-gradient AS is usually caused by the presence of a low LV outflow condition, which can occur with reduced LVEF, i.e., either classic (low LVEF) low-flow/low-gradient AS or paradoxical (preserved LVEF) low-flow/low-gradient AS (4). Paradoxical low-flow/low-gradient AS is a severe form of AS characterized by low cardiac output and low transvalvular gradient and has a poor prognosis (2). Reduced cardiac output could be due to reduced LVEF or excessive cardiac remodeling and/or restrictive physiology with preserved LVEF. Dobutamine stress echocardiography is used to confirm AS severity (peak stress mean gradient ≥40 mmHg) in patients with low-flow/low-gradient AS and reduced LVEF (1, 5).

Although severe AS is associated with a poor prognosis, surgical (SAVR) or transcatheter (TAVR) AV replacement can restore a patient's life expectancy to that of the age- and sex-matched population (1).

Cardiac amyloidosis (CA) is a serious progressive disease that results from the infiltration of misfolded protein fragments into the cardiac muscle and is characterized by extracellular deposits of amyloid fibrils in the myocardium and other cardiac tissues, resulting in LV dysfunction (1, 6). As with AS, the prevalence of CA increases with age, and CA has been estimated to affect almost 25% of individuals aged ≥80 years (1). Of these individuals, the vast majority are neither suspected of having CA nor systematically tested for CA during their management (7). The most common CA types are light chain (AL) amyloidosis, caused by monoclonal immunoglobulin light chains, and transthyretin amyloidosis (ATTR-CM), caused by either mutated or wild-type transthyretin protein aggregates (1, 8).

CA is an underrecognized cause of common cardiac conditions, including HF with preserved ejection fraction (HFpEF) or HF with mildly reduced ejection fraction (HFmrEF) (6, 9, 10). Advances in imaging techniques and the possibility of non-invasive diagnosis have revealed CA to be a more frequent disease than previously believed (6).

CA is increasingly diagnosed in patients who may be misdiagnosed as having undifferentiated HFpEF, paradoxical low-flow/low-gradient AS, or otherwise unexplained LV hypertrophy (8). CA has been recognized in the validated HFA-PEFF diagnostic algorithm as an important treatable cause of HFpEF. Specific diagnostic tests such as scintigraphy, cardiovascular magnetic resonance (CMR), and endomyocardial biopsy have been recommended in this diagnostic algorithm for confirmed HFpEF patients with high-risk features of CA (11, 12).

TAVR rather than SAVR may be preferred in patients with CA and is increasingly becoming the standard of care among structural heart disease teams (13). For patients with a confirmed ATTR-CM diagnosis and, preferably, New York Heart Association (NYHA) Functional Classification Class I or II, pharmacological treatment should be initiated (1).

### 1.2 Relationship between AS and CA

Retrospective and prospective studies indicate the prevalence of CA in patients with AS ranges between 8% and 16% (14, 15), with ATTR-CM being the most prevalent form (16). CA negatively affects the outcome of patients with AS, and patients with both AS and CA have a significantly higher overall mortality rate than those with AS alone (15). Treatment with CA pharmacotherapy can be expected to have a significant outcome in patients with combined AS and ATTR (15).

Similarly to CA patients, AS patients often exhibit significant LV hypertrophy, which can be a confounding factor. Compared with patients who have AS alone, those with concomitant AS and CA are older, have worse functional status, worse cardiac remodeling, higher circulating N-terminal probrain natriuretic peptide (NT-proBNP) and troponin levels, and more frequently exhibit a pattern of low-flow/low-gradient AS (1, 2, 14). After an assessment is made by a heart team, it is these comorbidities that would likely lead to a decision in favor of TAVR over SAVR. The coexistence of CA contributes to a patient's increased frailty and worse cardiac hemodynamics (14).

The presence of a low-flow/low-gradient pattern with preserved or mildly reduced EF, small valve area, severe concentric LV remodeling, restrictive filling pattern, unexpectedly significant systolic pulmonary hypertension, and right ventricle (RV) dysfunction in an elderly patient should raise a clinical suspicion of cardiac amyloidosis (1, 2), particularly in the presence of electrical conduction disturbances and atrial fibrillation (AF) (2).

Despite evidence that up to 16% of AS patients have concomitant CA, there is a lack of awareness among interventional cardiologists and heart teams on the association of AS and CA resulting in a lack of systematic testing for this statistically common association of diseases (1). Both AS and CA coexist in older adults and share several clinical and echocardiographic features. These common features, combined with AS independently leading to myocardial hypertrophy, may present confusion in the minds of physicians and prevent them from making the correct diagnosis (1, 9, 17, 18). The assumption that the conditions of LV hypertrophy and heart failure can be explained by the degree of AS in a patient may lead to an underdiagnosis of CA. This additional diagnosis of CA is often not considered because it is deemed to be a rare or untreatable condition, despite evidence to the contrary (1).

Although both AS and CA share pathological features, the prognosis for CA is usually worse than that for severe AS alone. The challenge for cardiologists is to identify “red flags” specific to CA in the AS population to raise a suspicion of dual diagnosis (14). The 2021 ESC Guidelines on the management of valvular heart disease highlight the high frequency of CA in elderly patients with AS and advocate for appropriate imaging for patients with amyloidosis (19). Currently, structural heart team discussions regarding elderly patients with AS typically focus on patient selection, comorbidities, and anatomical features (particularly CT scan and ECHO [echocardiogram]) to determine the feasibility of SAVR vs. TAVR rather than considering amyloidosis as noted by the guidelines.

### 1.3 Diagnosis

Given its negative effect on AS prognosis, the identification of CA in patients with AS is important (15). Successful management begins with a screening and suspicion of suspected CA in the AS population, followed by advanced diagnostic evaluation to confirm the diagnosis and then typing of the amyloid fibrils (8).

Historically, echocardiography, particularly the global strain assessment, has permitted the possible identification of CA and has been the initial testing technique in the diagnostic pathway. However, classical echocardiographic findings are somewhat nonspecific and may be absent at an early stage of the disease (9, 20). The advent of bone scintigraphy makes an early, non-invasive diagnosis of ATTR-CM possible, thereby averting the need for an endomyocardial biopsy (8, 16). Bone scintigraphy has both high sensitivity and high specificity to allow the identification of cardiac ATTR deposits early in the course of the disease, sometimes even before abnormalities are seen on echocardiography or cardiac magnetic resonance imaging (MRI) (9, 21). In all patients with suspected CA, the laboratory assessment should include analysis for the presence of a monoclonal immunoglobulin through the use of serum and urine immunofixation the quantification of serum-free immunoglobulin light chains, which are associated with AL (8).

Diagnostic tests for confirmation of CA, including bone scintigraphy (for ATTR-CM), serum/urine-free light chain assay (to rule out AL amyloidosis), and extracardiac tissue biopsy (in certain scenarios), should be considered in AS patients presenting with “red flags” for CA (Table 1).

**Table 1:** Red flags that raise the suspicion of CA (1, 14, 16, 22).

Diagnostic	Red flags
Patient history	Carpal tunnel syndrome (bilateral), lumbar spinal stenosis, and/or deafness in an elderly (≥65 years) male patient
	Family history of neuropathy or sudden cardiac death
	HF with a preserved ejection fraction
	Disproportionate HF symptoms
	Natural cure for hypertension
	Complaints of sensory peripheral neuropathy, foamy urine, and/or bleeding
ECG	Low QRS voltage or disproportionately low voltage in the presence of increased LV wall thickness/LV hypertrophy
	Conduction abnormalities (RBBB and/or AV block); atrial fibrillation
	Pseudo-infarct pattern (Q waves) in the absence of wall motion abnormalities on echocardiography
Echocardiography	Low-flow/low-gradient AS
	LV (and RV) hypertrophy
	Preserved LVEF, but reduced GLS with apical sparing
	Myocardial granular sparkling
	Atrial septal thickening, biatrial dilation
	Low QRS voltage to LV mass ratio
Cardiac MRI	Increased LV mass
	Transmural or subendocardial LGE not related to a coronary artery territory, diffuse atrial LGE, RV LGE, suboptimal nulling
	Increased myocardial native T1 values, increased extracellular volume, myocardial edema (T2)
Laboratory tests	Disproportionately high level of NT-proBNP
	Chronically elevated troponin at a low level with normal CAG

AV, atrioventricular; CAG, coronary angiography; ECG, electrocardiogram; GLS, global longitudinal strain; LGE, late-gadolinium enhancement; LV, left ventricle; LVEF, left ventricular ejection fraction; MRI, magnetic resonance imaging; NT-proBNP, N-terminal pro brain natriuretic peptide; RBBB, right bundle branch block; RV, right ventricle.

### 1.4 Suspicion criteria

Given that CA shares several features with AS, the challenge for cardiologists is to identify, in the AS population, clinical, demographic, electrocardiographic, echocardiographic, imaging, and laboratory “red flags” that suggest the coexistence of AS and CA (14). In addition to the red flags for the presence of CA listed in Table 1, a remodeling, age, injury, systemic, and electrical (RAISE) score ≥2, poor response to HF medications, and rapid progression to pacemaker implantation are also suggestive of CA involvement in AS patients. As with the other red flags, these are suggestive of CA but not sufficient to confirm the diagnosis.

### 1.5 RAISE score

The RAISE score is a validated clinical scoring system that integrates certain red flags to create an additional screening tool for CA in patients with AS. Nitsche et al. conceived the RAISE score to discriminate between patients with AS alone and those with concomitant CA (Table 2) (3). This score was derived from a large cohort of TAVR and SAVR patients who underwent systematic assessment for concomitant CA through the use of biomarkers and scintigraphy, which allows for a more selective approach to cardiac scintigraphy and CA testing (3).

**Table 2:** Five domains of the RAISE score, a screening tool for CA in patients with AS (total score ≥2 points suggests the presence of CA) (3).

Domain	Factor	Weighting
**M**yocardial **R**emodeling	Marked LV hypertrophy; septal wall thickness ≥18 mm	1 point
	Marked diastolic dysfunction, *E/A* ratio >1.4	1 point
**A**ge	≥85 years	1 point
**M**yocardial **I**njury	High-sensitivity troponin T	1 point
**S**ystemic **D**isease	Carpal tunnel syndrome	3 points
**E**lectrical **A**bnormalities	Right bundle branch block	2 points
	Low voltages/Sokolow–Lyon index <1.9 mV	1 point

*E/A*, ratio of peak velocity blood flow from LV relaxation in early diastole (the *E* wave) to peak velocity flow in late diastole caused by atrial contraction (the *A* wave); LV, left ventricle.

Based on consideration of five domains, a RAISE score ≥2 points indicates the presence of CA in patients with AS (3), prompting further assessment with bone scintigraphy and free light chain analysis. The RAISE score demonstrated high sensitivity and adequate specificity for the presence of CA in an AS cohort in which most patients with CA had ATTR. Scores ≥2 and ≥3 were found to have high sensitivity (93.6% and 72.3%) and adequate specificity (52.1% and 83.6%) (3). However, the need for further validation has been suggested (14).

### 1.6 Conduction system abnormalities and unanticipated pacemaker implantation

Unanticipated pacemaker implantation should be a suspicion criterion for CA in patients with AS (1, 16). Amyloid infiltration into the cardiac conduction system causes a range of electrophysiological disturbances, including AV nodal disease, which is common in CA, as is the requirement for a pacemaker (23). In a retrospective cohort study of patients with ATTR-CM, 9.5% had pacemakers implanted for a high-grade AV block prior to their diagnosis of CA, and another 11% underwent the process subsequent to their diagnosis (24). Compared with patients with HFpEF without a diagnosis of CA, those with CA and HFpEF require pacemakers significantly more frequently (23).

### 1.7 Assessment of the myocardium

While echocardiography is an essential first-line diagnostic tool that raises a suspicion for CA, cardiac MRI has an important diagnostic role in the workup of CA (25). Cardiac MRI enables a more comprehensive investigation of CA via high-resolution imaging, functional assessment, and superior tissue characterization (25). However, it appears to be underutilized. In a review of the association between AS and CA (16), cardiac MRI was used in only four of 13 published studies in which the imaging features of patients with both AS and CA were assessed. This observation suggests that the diagnostic focus is predominantly on the valve rather than on the myocardium. Similarly, in the European Society of Cardiology's Cardiovascular Imaging Toolboxes, the emphasis of the Multimodality Imaging Toolkit for AS is on AV morphology and flow evaluation rather than specific assessment of the myocardium (26). A classification of AS was published, in which not only the degree of valve damage but also the LV and myocardium damage are considered (27). According to this classification, patients with both CA and AS have a worse prognosis than at other stages of the classification (27). This classification system was confirmed in a large multicenter cohort of symptomatic patients with severe AS (28). In the 2021 ESC Guidelines for the Management of Valvular Heart Disease, CMR is emphasized for assessment of myocardial fibrosis and CA, although it is underutilized in clinical practice (19).

Although LV wall hypertrophy is expected in patients with AS, it should not be assumed to be caused only by AS, particularly in patients in whom the degree of hypertrophy is disproportionate to the degree of AS severity. Indeed, the accumulation of amyloid fibrils in the myocardium leads to progressive ventricular wall thickening and stiffness (22). Hence, cardiologists should consider the possibility of CA in their patients with AS and be proactive in investigating CA, especially during their assessment of the myocardium.

### 1.8 Treatment of concomitant AS and CA

With increasing confidence in TAVR and changes in guideline recommendations, analyses of European, UK, and US registries show a steady increase in the number of TAVR procedures performed (29). A high prevalence of CA has been observed in TAVR cohorts, with CA being found in approximately one-third of AS patients undergoing TAVR (16). This observation suggests that AS patients referred for TAVR should be systematically screened if there is any suspicion of coexistent CA.

Patients with concomitant CA and AS are likely to benefit from AV replacement. TAVR has been demonstrated to significantly improve the prognosis of patients with both AS and CA, with a survival rate similar to that in patients with AS alone (15). However, the presence of CA could be a factor in the choice of valve replacement procedure (SAVR vs. TAVR) for patients with AS.

Some studies have suggested better outcomes with TAVR than with SAVR in patients with AS and CA (1, 16). SAVR has been associated with a higher risk of several periprocedural complications (1, 16). When compared with medical therapy, the risk of mortality is lower with TAVR in patients with concomitant AS and CA (odds ratio 0.23; *P* = 0.001), and the safety profile of TAVR appears to be similar in patients with both AS and CA as compared with patients with AS alone (30). Therefore, it seems reasonable to prefer TAVR over SAVR, particularly given patients with AS and CA are often older and have a higher surgical risk score.

Data from the ATTR-ACT clinical trial and the ATTR-ACT long-term study demonstrated that tafamidis treatment significantly improved long-term outcomes in patients with CA (31, 32). Early and continuous treatment with tafamidis (up to 72 months) showed a significant 41% reduction in mortality (*P* < 0.001) and a 44% improvement in NYHA Class (*P* = 0.003) (32). This highlights the need for a multidisciplinary team to discuss individual cases of concomitant AS and CA to select the best treatment options.

## 2 Screening for AS and CA: case study examples

### 2.1 Case study 1

#### 2.1.1 Presentation

A 101-year-old man (**A**) presented with progressively worsening lower-extremity edema and associated shortness of breath. Recently, he noted an increasing lower-extremity swelling and open skin wounds on the left leg with clear drainage and an increasing shortness of breath. Comorbidities included spinal stenosis, type 2 diabetes with chronic kidney disease, hyperlipidemia, hypertension, and diabetic retinopathy. He reported no chest pain, dizziness, or syncope. He had a previous history of hospital admission due to respiratory failure. He had been diagnosed with a left upper lobe lung mass, severe AS with concentric LV hypertrophy (**R**), and an EF of 40% with grade 3 diastolic dysfunction.

#### 2.1.2 Investigations

**Figure 1:**
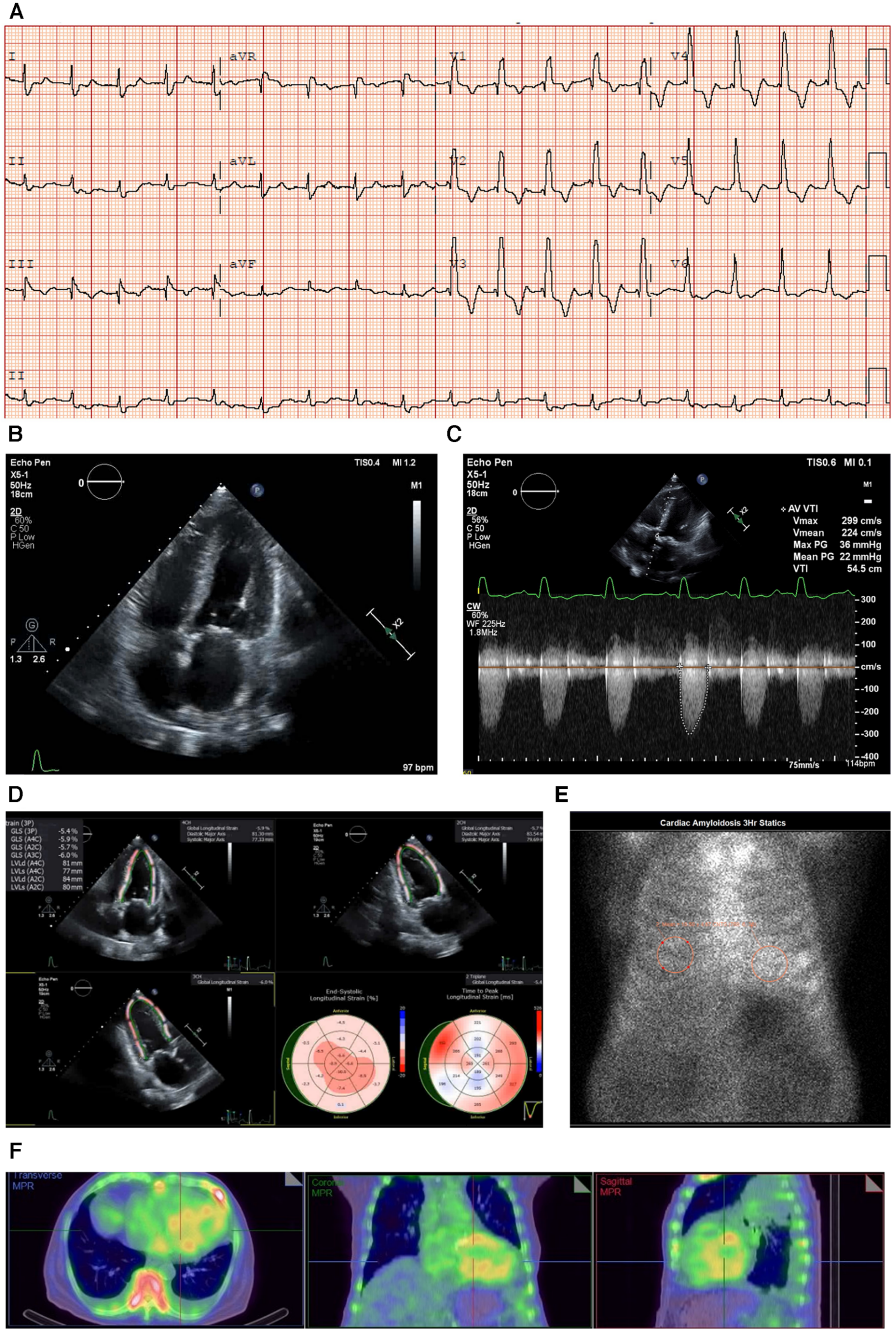
Case 1 clinical investigations. (**A**) An ECG showing sinus rhythm with first-degree heart block and RBBB morphology, and corrected QT interval prolonged for a heart rate of 98 bpm. (**B**) A 2D echocardiography with a four-chamber view showing LV hypertrophy with biatrial dilatation. (**C**) Continuous-wave Doppler of the AV consistent with low-flow/low-gradient AS. The calculated AV area was 0.47 cm^2^. (**D**) An echocardiography GLS showing markedly reduced GLS with apical sparing, typical of a diagnosis of CA. (**E**) A planar bone scintigraphy (^99m^Tc-PYP) scan at 3 h with a H/CL ratio of 1.33. (**F**) SPECT-CT fusion showing an intense myocardial uptake with a clear LV cavity, excluding the presence of a blood pool. AS, aortic stenosis; AV, aortic valve; CA, cardiac amyloidosis; ECG, electrocardiogram; GLS, global longitudinal strain; H/CL, heart to contralateral lung; LV, left ventricular; RBBB, right bundle branch block; SPECT-CT, single-photon emission computed tomography; ^99m^Tc-PYP, technetium pyrophosphate.

An electrocardiogram (ECG) showed sinus rhythm with a first-degree heart block and right bundle branch block (RBBB) morphology, and a corrected QT interval prolonged for a heart rate of 98 bpm (**E**) (Figure 1A).

An echocardiogram revealed concentric LV hypertrophy (interventricular septum of 1.3 cm; posterior wall measuring 1.4 cm; a relative wall thickness of 0.7, demonstrating significant hypertrophy) with an EF of 33 ± 5% and a moderate biatrial dilatation (**R**) (Figure 1B). Mild mitral stenosis was observed, and the mean mitral valve gradient was 4 mmHg. Severe AV stenosis was caused by a calcified valve, and the AV area was 0.47 cm^2^ (Figure 1C). The peak gradient was 40 mmHg and the mean gradient was 25 mmHg, demonstrating low-flow/low-gradient AS. The stroke volume index (SVi) was 17.2 ml/m^2^ and the *E*/*A* ratio was 3 (**R**). Red flags seen on the echocardiogram included atrial septal thickening and a markedly reduced global longitudinal strain of −5.41 with relative apical sparing (Figure 1D).

Due to the strong clinical suspicion for CA, a ^99m^Tc-PYP scan was performed, which demonstrated a heart-to-contralateral lung (H/CL) ratio of 1.33 at 3 h (Figure 1E). Single-photon emission computed tomography (SPECT-CT) scans revealed an intense myocardial uptake (Figure 1F).

The laboratory results were as follows: BNP 1,448.4 ng/L, NT-proBNP 10,476 ng/L, urea 21.9 mmol/L, creatinine 182 µmol/L, urine albumin/creatinine ratio 44.98 mg/mmol, and estimated glomerular filtration rate (eGFR) 33 ml/min/1.73 m^2^, which demonstrated that the patient had moderate renal failure.

Serum immunofixation studies and free light chain analysis were performed. A marked elevation of the serum-free kappa and lambda light chains was noted (126.04 and 88.87 mg/L, respectively); however, the ratio was within the accepted range for renal impairment. The troponin T level was 0.56 μg/L (reference range 0.00–0.05 μg/L) (**I**).

#### 2.1.3 Treatment and interpretation

In this elderly patient with severe AS, the diagnosis was late-stage CA with HF that had progressed to reduced EF and severe calcific AS, making him eligible for TAVR and concomitant treatment with CA therapies. Treatment with TAVR was discussed at a meeting of the structural heart team, but given the patient's age (101 years) and comorbidities, his family did not give consent for TAVR; however, the patient was started on tafamidis (61 mg once daily). Unfortunately, seven months after the initiation of treatment, the patient passed away due to complications from pneumonia. Statistically speaking, for patients with AS in this age group, wild-type ATTR would be a more likely diagnosis. However, at the time of evaluation, genetic testing was not covered by insurance, and given the patient’s age, his family declined to pursue the option of commercial genetic testing. Genetic testing would not influence the treatment decision for TTR cardiac amyloidosis.

RAISE score total: 6.

### 2.2 Case study 2

#### 2.2.1 Presentation

This patient case relates to a woman aged 81 with a history of HFpEF (50% EF), hypertension, hyperlipidemia, moderate AS, moderate tricuspid regurgitation, pulmonary hypertension, and chronic AF treated with apixaban and metoprolol. She was also receiving treatment with furosemide, spironolactone, and valsartan. Because of an episode of hypoxia with a loss of consciousness, she was admitted to the ICU. A chest x-ray showed a large right-sided pleural effusion. The patient showed improvement with non-invasive ventilation and diuresis, but within a week, she was seen to have increased somnolescence and hypoxia. She required bilevel positive airway pressure (BiPAP) because of hypercarbia and received a furosemide infusion, to which she showed a good response.

#### 2.2.2 Investigations

**Figure 2:**
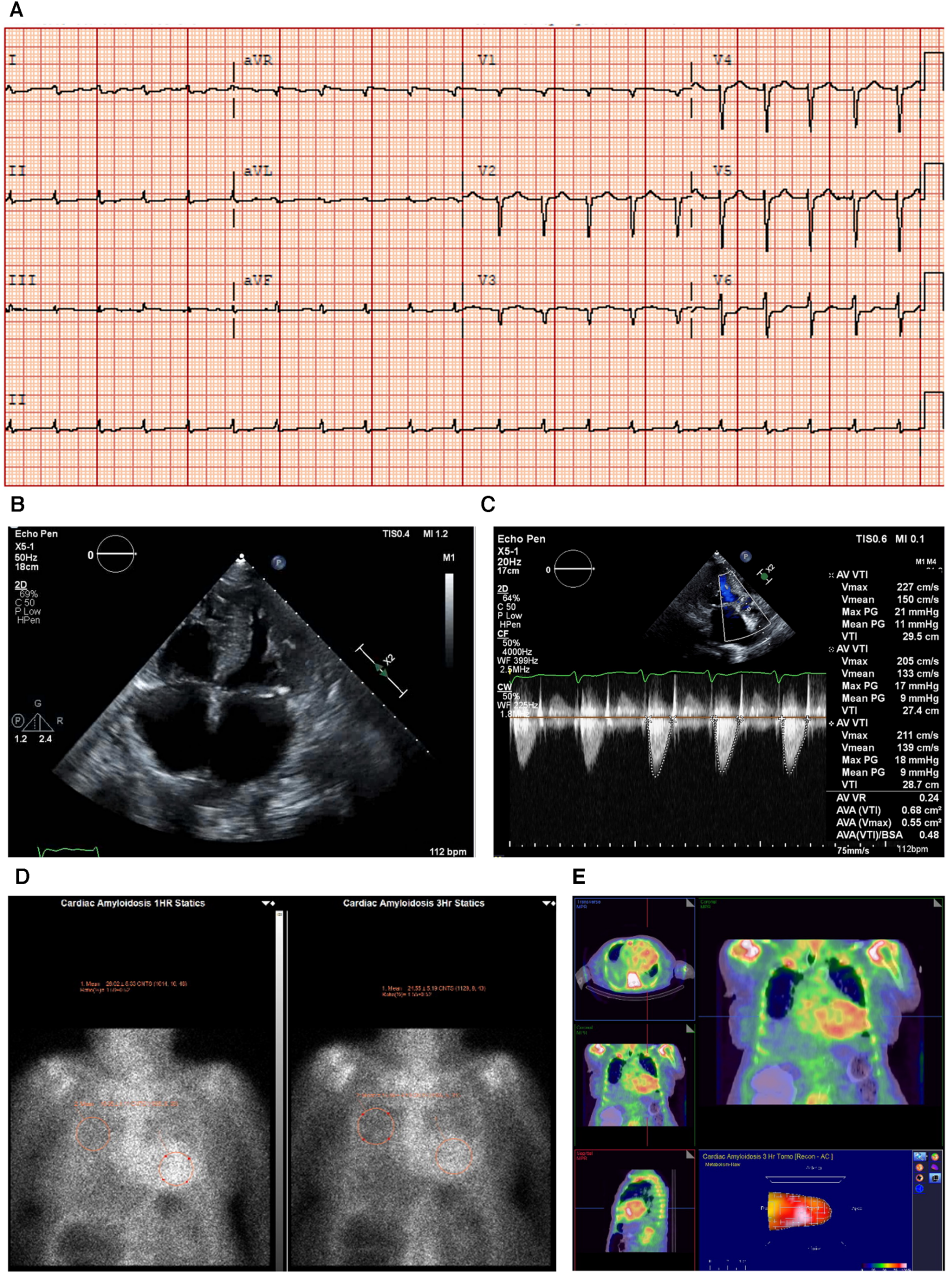
Case 2 clinical investigations. (**A**) An ECG showing sinus rhythm, low-voltage limb leads, poor R-wave progression, and a pseudo-infarct pattern. (**B**) A 2D echocardiography with a four-chamber view showing marked concentric LV hypertrophy and biatrial enlargement. (**C**) Continuous-wave Doppler of the AV is consistent with paradoxical low-flow/low-gradient. The calculated AV area index was 0.45 cm^2^/m. (**D**) Planar bone scintigraphy (^99m^Tc-PYP) scan at 1 h and 3 h showing grade 3 myocardial uptake. (**E**) A SPECT scan demonstrating a strongly positive uptake (grade 3) with clear-cut differentiation between the LV cavity and myocardial uptake. AV, aortic valve; ECG, electrocardiogram; LV, left ventricular; SPECT, Single-photon emission computed tomography; ^99m^Tc-PYP, technetium pyrophosphate.

An ECG showed sinus rhythm, low-voltage limb leads (**E**), poor R wave progression, and a pseudo-infarct pattern. As is to be expected in a patient with CA, the typical ECG pattern revealed discordance between low ECG voltage and LV hypertrophy on the ECHO (Figure 2A) (33).

An echocardiography demonstrated a small left ventricle with severe concentric LV hypertrophy (**R**). LV systolic function was mildly decreased. The EF was estimated at 50 ± 5% with resting wall motion abnormalities. The right ventricle was normal in size, but RV systolic function was moderately to severely decreased [RV fractional area change, 22%; RVEF, 27%; tricuspid annular plane systolic excursion (TAPSE), 9 mm; RV basal diameter, 38 mm] (Figure 2B). The left and right atrial cavities were severely dilated. There was a severe AV stenosis caused by a calcified valve and a restricted opening. The overall findings were consistent with paradoxical low-flow/low-gradient severe AS. The SVi was 12 ml/m^2^, AV area index was 0.45 cm^2^/m, peak gradient was 17 mmHg, and mean gradient was 8 mmHg (Figure 2C). The *E*/*A* ratio could not be obtained as the patient had chronic AF.

Due to the strong clinical suspicion of CA, a ^99m^Tc-PYP scan was conducted (Figure 2D). In semiquantitative assessment, myocardial PYP uptake was higher than rib uptake (grade 3). At 1 and 3 h SPECT studies, no evidence of excess blood pool was noted in the LV cavity (Figure 2E). At 1 h, the H/CL ratio was 1.61, and at 3 h, the H/CL ratio was 1.55.

Serum and urine immunofixation presented an apparent normal pattern, demonstrating that this patient had ATTR-CM. The troponin T level was 0.193 μg/L (reference range <0.06 μg/L) (**I**).

#### 2.2.3 Treatment and interpretation

Treatment with tafamidis (61 mg once daily) was initiated in November 2020, and the patient remains under treatment to date. TAVR was offered to the patient, but she declined surgery. This case clearly highlights the coexistence of severe AS with LV hypertrophy due to CA that is consistent with the ECG parameters and provides a pathophysiological explanation for the low-flow/low-gradient form of AS. While AS itself results in LV hypertrophy, it should not result in low-voltage ECG, thus increasing the suspicion of two concomitant disorders (15).

RAISE score total: 3.

### 2.3 Case study 3

#### 2.3.1 Presentation

An 85-year-old man (**A**) presented with no significant cardiovascular risk factors but complained of shortness of breath and dyspnea upon exertion with progressive aggravation during the past year. A clinical examination revealed a harsh systolic murmur, no signs of pulmonary edema, a regular heart rate of 82 bpm, and blood pressure of 110/70 mmHg.

#### 2.3.2 Investigations

The laboratory tests demonstrated the following: eGFR 45 ml/min, potassium 4 mEq/L, NT-proBNP 3200 ng/L, and hemoglobin 11.5 g/dl. A troponin test was not performed.

**Figure 3:**
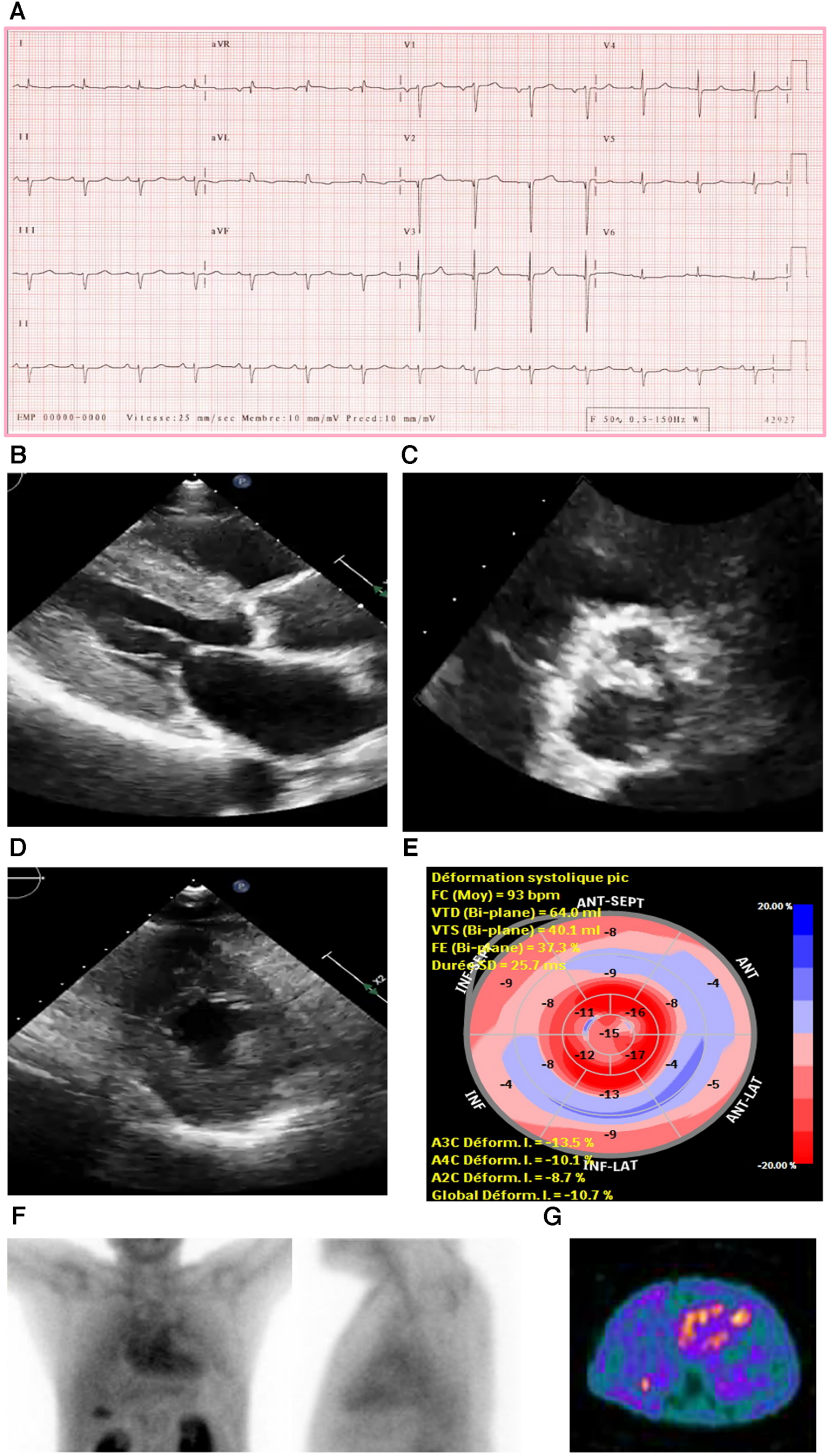
Case 3 clinical investigations. (**A**) An ECG showing regular sinus rhythm with low voltage in the limb leads and lateral leads with a prolonged PR interval (240 ms). A Q wave in v2 and v3 is noted. (**B–D**) A 2D echocardiography demonstrating marked concentric LV hypertrophy with severely calcified trileaflet AV causing significant AS, with low-flow and low-gradient. The EF is reduced. (**E**) Apical sparing with GLS reduction is seen. (**F**) An anterior and lateral bone scintigraphy (^99m^Tc-PYP) scan at 1 h showing grade 3 myocardial uptake. (**G**) A SPECT scan demonstrating strongly positive uptake (grade 3). AS, aortic stenosis; AV, aortic valve; ECG, electrocardiogram; EF, ejection fraction; LV, left ventricular; SPECT, Single-photon emission computerized tomography; ^99m^Tc-PYP, technetium pyrophosphate.

An ECG showed a regular sinus rhythm, narrow QRS, low voltage in peripheral leads (**E**), and q waves mainly in V2 and V3 (Figure 3A).

An echocardiography demonstrated a small LV cavity and moderate concentric LV hypertrophy (**R**). There was mild to moderate global hypokinesis, with the EF calculated at 35%–40% (Figures 3B–E). The global longitudinal strain (Figure 3C) was moderately reduced and measured at −10.7% with a pattern of relative apical sparing and a ratio of basal strain segments/apical strain segments of 2. Diastolic parameters demonstrated moderate diastolic dysfunction with an *E*/*A* ratio of 1.4 consistent with a pseudo normal pattern and a moderately raised LV filling pressure. The patient exhibited a severely calcified trileaflet AV causing a significant low-flow/low-gradient AS. The SVi was 31 ml/m^2^, AV area was 1.09 cm^2^, peak gradient was 24 mmHg, and mean gradient was 14 mmHg. The patient had moderate pulmonary hypertension, and both atria were moderately dilated with high right atrial and systolic pulmonary pressure.

Due to these electrical and echocardiographic findings, there was a strong suspicion of coexistent CA in this patient with severe AS. Therefore, a bone scintigraphy of the heart was conducted (Figures 3F,G), which showed strong tracer uptake within the myocardium (grade 3) and a H/CL ratio of 2.0. A hematological workup was performed and was negative for the presence of any monoclonal component with a normal free light chain ratio, ruling out AL amyloidosis (serum and urine immunofixation test result was negative, and serum free light chain assay was in the normal range: kappa 12.5 mg/L and lambda 20.8 mg/L).

#### 2.3.3 Treatment and interpretation

This patient had clinical signs of HF caused by low-flow/low-gradient severe AS alongside ATTR-CM. No additional tests, such as cardiac or extracardiac biopsy were performed since the positive predictive value (PPV) of PYP bone scintigraphy of the heart was 100% in the absence of monoclonal gammopathy. The heart team advised TAVR, but both the patient and his family declined it. Instead, the patient was medically treated to alleviate his symptoms.

RAISE score total: 4.

### 2.4 Case study 4

#### 2.4.1 Presentation

A 69-year-old man presented with a known history of coronary artery disease, a previous percutaneous coronary intervention, AS, a second-degree Mobitz, hypertension, hyperlipidemia, cervical and spinal stenosis, type 2 diabetes, chronic kidney disease, benign prostatic hypertrophy, and vitamin D deficiency. He was experiencing episodes of orthostatic dizziness while standing, accompanied by chest pain and dyspnea upon exertion, which he described as a burning sensation that relieves when he walked. He had mild ankle edema.

#### 2.4.2 Investigations

**Figure 4:**
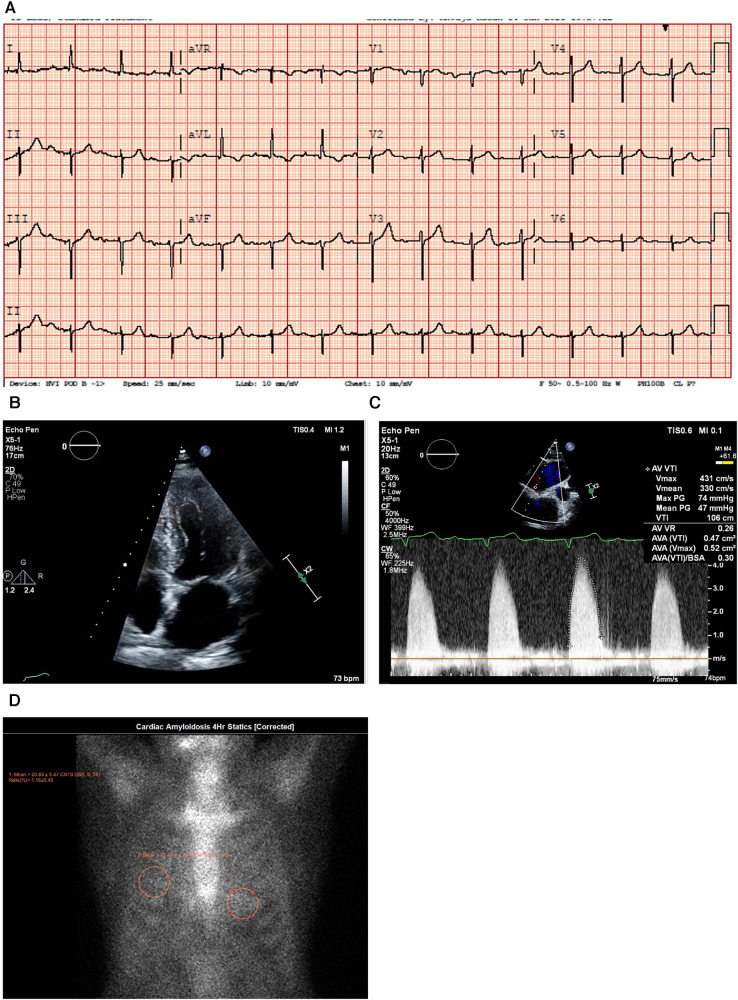
Case 4 clinical investigations. (**A**) An ECG showing normal sinus rhythm with prolonged PR interval, 360 ms first-degree AV block, T-wave inversion in aVL, and left-axis deviation with a left anterior vesicular block. (**B**) A 2D echocardiography with four-chamber view showing LV hypertrophy with a dilated left atrium and thickened atrial septum. (**C**) Continuous-wave Doppler of the AV consistent with AS. The calculated AV area was 0.5 cm^2^. (**D**) A planar bone scintigraphy (^99m^Tc-PYP) scan at 4 h showing myocardial uptake that is less than rib uptake (grade 1). AV, aortic valve; ECG, electrocardiogram; LV, left ventricular; ^99m^Tc-PYP, technetium pyrophosphate.

An ECG demonstrated normal sinus rhythm with prolonged PR interval, a 360 ms first-degree AV block, T-wave inversion in the aVL (augmented Vector Left), and left axis deviation with left anterior vesicular block (Figure 4A).

An echocardiogram (Figure 4B) highlighted a normal-sized left ventricle with mild concentric LV hypertrophy (**R**). The LV systolic function was normal, and the EF was 64 ± 5%. The global LV myocardial strain was normal, and LV diastolic function was grade II. The left atrial cavity was mildly dilated, and the patient had mild to moderate mitral regurgitation. The right ventricle was normal in size and RV systolic function was normal. The *E/A* ratio was 1.0. Severe AV stenosis was demonstrated with a peak velocity of 4.3 m/s, a mean gradient of 47 mmHg, an AV area of 0.47 cm^2^, and an SVi of 32 ml/m^2^ (Figure 4C).

A hematological workup was performed, and the serum and urine immunofixation test results did not show monoclonal gammopathy. The serum kappa/lambda ratio was 2.06 and was determined to be negative for AL amyloidosis.

A PYP scan demonstrated that myocardial PYP uptake was less than rib uptake (grade 1), and SPECT-CT images did not demonstrate evidence of myocardial tracer uptake (Figure 4D).

#### 2.4.3 Treatment and interpretation

A clinical suspicion of CA was raised in this patient. However, the result of the PYP scan was negative for CA, and therefore, the patient underwent treatment with an uncomplicated TAVR. A retrospective application of the RAISE score (Table 3) demonstrated that the patient would not have aroused suspicion for CA, and therefore, the PYP scan need not have been performed.

**Table 3:** Evaluation of case studies against the RAISE score.

Domain	Factor	Case 1	Case 2	Case 3	Case 4	Case 5
Myocardial **R**emodeling	Marked LV hypertrophy; septal wall thickness ≥18 mm	✓	✓	✓	✓	✓
	Marked diastolic dysfunction, *E/A* ratio >1.4	✓	X^a^	✓		X^a^
**A**ge	≥85 years	✓		✓		✓
Myocardial **I**njury	High-sensitivity troponin T	✓	✓	Not measured		
**S**ystemic disease	Carpal tunnel syndrome					✓
**E**lectrical abnormalities	Right bundle branch block	✓				X^b^
	Low voltages/Sokolow–Lyon index <1.9 mV		✓	✓		
Total Points		6	3	4	1	6

*E*/*A*, ratio of peak velocity blood flow from LV relaxation in early diastole (the *E* wave) to peak velocity flow in late diastole caused by atrial contraction (the *A* wave); LV, left ventricle.

^a^ The patient has chronic atrial fibrillation, and therefore, *E/A* cannot be obtained.

^b^ The patient has a paced rhythm, and therefore, a right bundle branch block cannot be ascertained.

RAISE score total: 1.

### 2.5 Case study 5

#### 2.5.1 Presentation

**Figure 5:**
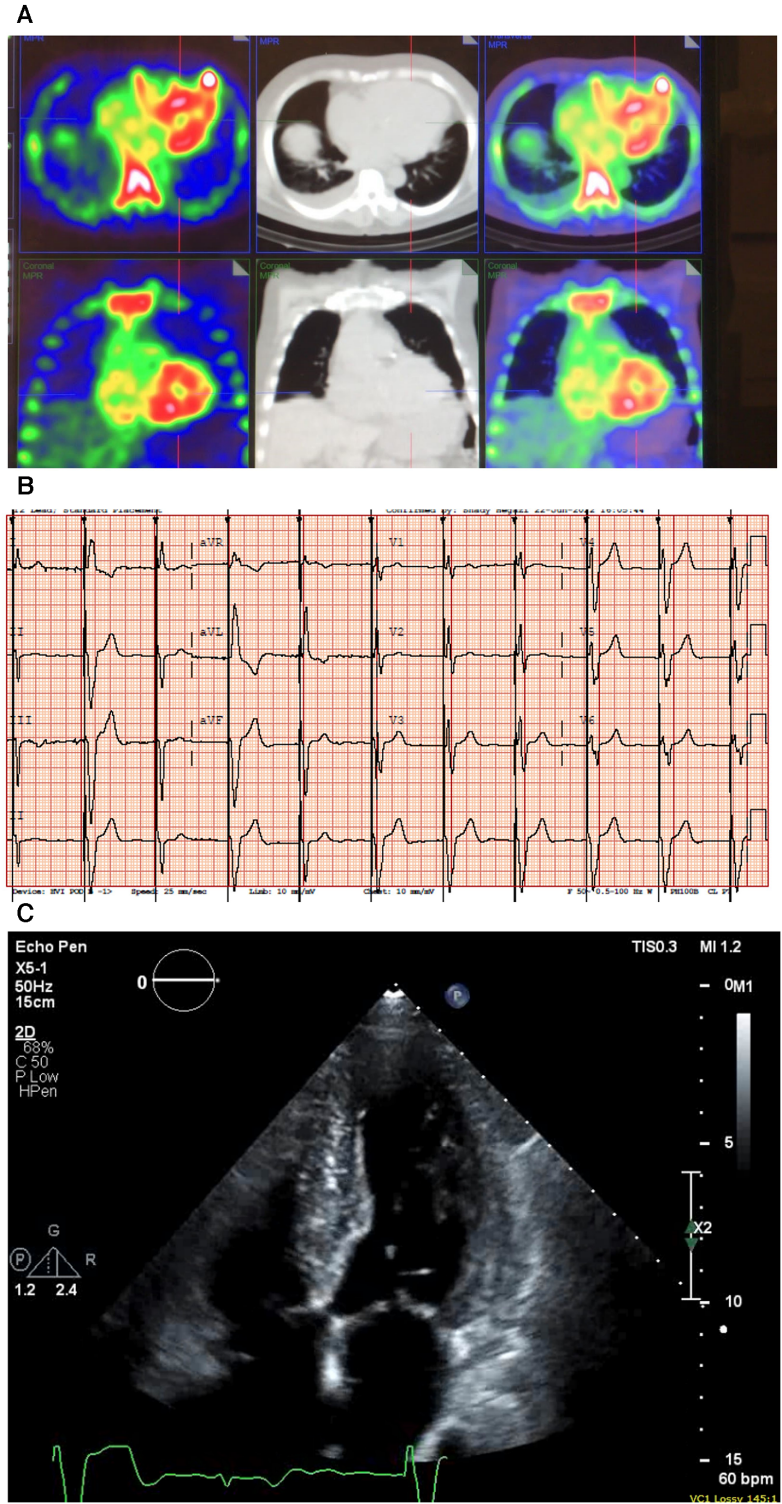
Case 5 clinical investigations. (**A**) A ^99m^Tc-PYP CT scan showing increased uptake in the myocardium suggestive of cardiac amyloidosis. (**B**) An ECG showing atrial-sensed ventricular-paced rhythm. (**C**) A 2D echocardiography with a four-chamber view showing concentric LV hypertrophy. CT, computed tomography; ECG, electrocardiogram; LV, left ventricular; ^99m^Tc-PYP, technetium pyrophosphate.

A 91-year-old (**A**) man presented with a history of HF, LV hypertrophy (**R**), carpal tunnel syndrome (**S**), spinal stenosis, and peripheral neuropathy. His previous workup included an echocardiograph that showed an EF of 47%, biatrial dilation, a marked LV hypertrophy (**R**), a septum of 1.4 mm, and a posterior wall thickness of 1.4, as well as grade III diastolic dysfunction. He had AS with a mean gradient of 10 mmHg, consistent with low-flow/low-gradient AS. He previously underwent a PYP scan that showed severe uptake (grade III) confirming a diagnosis of CA (Figure 5A), and his hematological test results were negative for AL amyloidosis. He was treated with diuretics and tafamidis.

#### 2.5.2 Investigations

An ECG demonstrated intermittent paced rhythm and a diffused low voltage (Figure 5B). An echocardiography showed severe concentric LV hypertrophy (**R**). LV systolic function was normal with an EF of 54 ± 5%, and the global LV myocardial strain was borderline abnormal. The RV was mildly dilated, and right ventricular systolic function was mildly decreased. The patient had mild AV stenosis; the SVi was >35 ml/m^2^, AV area was 1.6 cm^2^, peak gradient was 22 mmHg, and mean gradient was 13 mmHg (Figure 5C). The laboratory investigations showed an NT-proBNP level of 1,913 mg/L and a hemoglobin level of 127 g/L. A hematological workup was performed, and the serum kappa/lambda ratio was 2.12, which was negative for AL amyloidosis.

#### 2.5.3 Treatment and interpretation

This patient had concomitant CA and AS and was initiated on treatment with tafamidis (61 mg once daily) in early 2022. He remains under treatment to date. In the evaluation of this case, it should be noted that due to the paced rhythm (VVI [ventricular demand pacing] at 60 bpm with no underlying native escape rhythm), neither low voltage nor RBBB could be detected on ECG.

RAISE score total: 6.

### 2.6 Case study evaluation

The five patient cases presented here were evaluated against the RAISE score and their points were tallied (Table 3). This evaluation clearly demonstrates that the patient in Case Study 4 does not have CA, and if the RAISE score had been applied, the bone scintigraphy scan could have been avoided. These cases provide further practical validation of the RAISE score in a real-world setting, demonstrating that for AS patients the RAISE score can be used to effectively screen for CA prior to conducting a bone scintigraphy scan. Familiarity with the RAISE score by heart teams would allow for systematic preprocedural evaluation of patients who are due to undergo TAVR/SAVR. Consistent application of the RAISE score in this population of patients would allow for appropriate selection of specific patients for whom scintigraphy should be recommended. While it may be tempting to suggest that all elderly patients undergoing TAVR/SAVR evaluation should have a bone scintigraphy scan, the systematic application of the RAISE score would allow for a more judicious selection of patients, as is clearly demonstrated the patient in Case Study 4.

## 3 Conclusions

CA is an underappreciated cause of HF, especially in older people. Retrospective and prospective studies indicate that the prevalence of CA in patients with AS ranges between 8% and 16% (14, 15), with ATTR-CM being the most prevalent form (16).

The coexistence of AS and CA presents a diagnostic challenge, hence red flags for underlying CA should be systematically incorporated into clinical practice. LV wall hypertrophy is expected in patients with AS; however, it should not be assumed to be caused only by AS, particularly in patients in whom the degree of hypertrophy is disproportionate when compared to the degree of AS severity. Accordingly, a diagnostic process that includes screening for clinical, electrical, and imaging red flags for CA use of the simplified RAISE score should be applied to all patients with AS. A score ≥2 should prompt non-invasive assessment with PYP scintigraphy of the heart, which has very high sensitivity, specificity, and PPV, after the exclusion of AL-CA. This is necessary to improve the management of AS and CA, especially given the benefits of AV replacement even in the presence of both diseases, and the availability of novel pharmacological treatments for CA.

Combining the benefits of TAVR and pharmacotherapy in patients with AS and CA would likely result in a significant improvement in patient outcomes. Therefore, prospective evaluation with the RAISE score at the time of TAVR planning should be part of the systematic assessment. Additionally, with the aim of modifying postprocedural therapy, a retrospective analysis of the TAVR/SAVR databases should be conducted for applying the RAISE score. This will allow for detection of patients with concomitant AS and previously unsuspected CA and lead to subsequent referral for bone scintigraphy and treatment.

Patients with AS and unsuspected CA are typically followed up in an interventional or structural heart disease clinic and not in an HF clinic. This lack of collaboration between interventional cardiologists and the structural heart team is a major unmet need.

## Data Availability

The original contributions presented in the study are included in the article/Supplementary Material, and further inquiries can be directed to the corresponding author.
