# Corrigendum: A wolf in sheep’s clothing—aortic stenosis and cardiac amyloidosis: “RAISE”ing awareness in clinical practice

**DOI:** 10.3389/fcvm.2024.1418216

**Published:** 2024-04-26

**Authors:** H. Sabbour, K. Al-Humood, Z. Al Taha, I. Romany, H. Haddadin, D. Mohty

**Affiliations:** ^1^ Heart and Vascular Institute, Cleveland Clinic, Abu Dhabi, United Arab Emirates; ^2^ Warren Alpert School of Medicine, Brown University, Providence, RI, United States; ^3^ Advanced Heart Failure and Transplantation Unit, Chest Disease Hospital, Kuwait City, Kuwait; ^4^ Sheikh Shakhbout Medical City, Abu Dhabi, United Arab Emirates; ^5^ Pfizer Gulf FZ LLC, Dubai, United Arab Emirates; ^6^ Heart Center, King Faisal Specialist Hospital, Riyadh, Saudi Arabia

**Keywords:** aortic stenosis, diagnosis, cardiac amyloidosis, low-flow/low-gradient, RAISE score, transthyretin, heart failure

A Corrigendum on A wolf in sheep’s clothing—aortic stenosis and cardiac amyloidosis: “RAISE”ing awareness in clinical practice; By Sabbour H, Al-Humood K, Al Taha Z, Romany I, Haddadin H and Mohty D (2024). Front Cardiovasc Med. 11:1323023. doi: 10.3389/fcvm.2024.1323023

In the published article, there was an error. A colleague was omitted from the acknowledgments. A correction has been made to the **Acknowledgments**. This section previously stated:

“The authors thank Laura D’Castro and Steve Holliday from Astraeus Medical Ltd for editorial support in the preparation of this manuscript and also thank Ahmed Osman, Pfizer Saudi Limited, for his review of the manuscript.”

The corrected section appears below:

“The authors are grateful to Dr Haluk Alibazoglu from Cleveland Clinic Abu Dhabi for the preparation of patient nuclear medicine images. The authors thank Laura D’Castro and Steve Holliday from Astraeus Medical Ltd for editorial support in the preparation of this manuscript and also thank Ahmed Osman, Pfizer Saudi Limited, for his review of the manuscript.”

The authors apologize for this error and state that this does not change the scientific conclusions of the article in any way. The original article has been updated.

